# Dataset on the elastic modulus of heat-set whey protein isolate/xanthan gum mixed biopolymer hydrogels filled with glass microspheres: A model particle-filled composite food system

**DOI:** 10.1016/j.dib.2019.104066

**Published:** 2019-05-27

**Authors:** Andrew J. Gravelle, Reed A. Nicholson, Shai Barbut, Alejandro G. Marangoni

**Affiliations:** Department of Food Science, University of Guelph, Guelph, ON, Canada

## Abstract

This article presents data related to the research article entitled ‘Considerations for readdressing theoretical descriptions of particle-filled composite food gels’ [1]. The elastic modulus of mixed biopolymer composite gels consisting of heat-set whey protein isolate and xanthan gum (WPI-X) filled with glass microspheres is reported. Gels were evaluated as a function of volume fraction filler (φ_f_ = 0–0.5), with varying filler size (4 μm, 7–10 μm, 45–90 μm, and 150–210 μm) and ionic strength of the protein phase (0, 50, 100, 200 mM NaCl). The reported elastic modulus data was extracted from large deformation uniaxial compression tests. This data is relevant to the development of alternative particle reinforcement models, or adaptation of existing theories. Further, it represents the limiting case of rigid inclusions, which can eliminate certain confounding assumptions in established models.

**Table:** Specifications Table

Subject area	*Agricultural and Biological Sciences*
More specific subject area	*Food Science, Materials Science*
Type of data	*Table, figure*
How data was acquired	*Large deformation analysis via uniaxial compression (TA.XT2, Stable Microsystems, Texture Technologies Corp., Scarsdale, NY, USA)*
Data format	*Analyzed*
Experimental factors	*WPI-X gels: 10 wt% protein, 0.8 wt% hydrocolloid; pH 7.5, gelation* 30 min *@ 80°C* *Compression rate - 0.*83 mm/sec*; final compression strain of 60%*
Experimental features	*Determination of elastic modulus for composite gels containing model rigid fillers*
Data source location	*Not applicable*
Data accessibility	*All data is available within this article*
Related research article	*A.J. Gravelle, R.A. Nicholson, S. Barbut, A.G. Marangoni, Considerations for readdressing theoretical descriptions of particle-reinforced composite food gels, Food Res. Int. **122**, 209-221,*https://doi.org/10.1016/j.foodres.2019.03.070

**Table:** 

**Value of the data** • Incorporating glass microspheres over a broad concentration range into heat-set whey protein isolate/xanthan gum (WPI-X) gels provides insight into the influence of fillers on the rheological properties of composite food gels. • The use of homogeneously dispersed, rigid spherical inclusions satisfies several fundamental assumptions required in most established particle reinforcement theories, and minimizes the number of confounding variables which must be addressed. • Increasing ionic strength of WPI-X gels weakened matrix/filler interactions, providing a means of evaluating imperfect interfacial adhesion. • This dataset could be used to assist with validating newly developed particle reinforcement models, or proposed modifications to existing theories.

## 1 Data

The data presented in this section contains tabulated values of the elastic constant of mixed biopolymer composite gels (E_c_) consisting of heat-set whey protein isolate and xanthan gum (WPI-X) filled with glass microspheres. The values of E_c_ presented in Table 1, Table 2, Table 3 are reported as a function of volume fraction filler content (φ_f_ = 0–0.5), and are separated based on the ionic strength (0, 100, and 200 mM added NaCl) and filler size employed (4 μm, 7–10 μm, 45–90 μm, and 150–210 μm). The addition of NaCl was used to induce changes in the microstructural organization and mechanical properties of the resulting gel [2] (also, see associated research article [1]).

**Table 1:** Elastic modulus of heat-set whey protein isolate/xanthan gum (WPI-X) gels prepared with 0 mM*NaCl*, and varying volume fraction filler (φ_f_). Glass microspheres of varying size were used as model fillers, which are denoted in the table.

φ_f_	4 μm	7–10 μm	45–90 μm	150–210 μm
0	6.95 ± 0.46	6.65 ± 0.52	13.63 ± 0.46	13.63 ± 0.46
0.025	10.44 ± 0.43	11.01 ± 0.54	16.03 ± 0.61	17.28 ± 0.53
0.05	12.45 ± 1.11	10.54 ± 0.33	19.14 ± 0.38	20.24 ± 1.52
0.10	27.22 ± 1.33	12.22 ± 0.27	25.33 ± 3.02	29.38 ± 4.57
0.15	30.01 ± 0.23	17.77 ± 0.97	37.21 ± 1.79	46.77 ± 11.30
0.20	46.52 ± 4.67	27.18 ± 2.64	76.87 ± 1.35	64.83 ± 6.14
0.33	128.58 ± 13.24	125.63 ± 10.00	252.69 ± 5.36	165.28 ± 19.88
0.43	436.86 ± 2.21	429.47 ± 59.85	601.06 ± 40.03	512.59 ± 18.77
0.50	1611.18 ± 62.75	865.73 ± 108.96	1247.22 ± 92.13	856.52 ± 9.10

**Table 2:** Elastic modulus of heat-set whey protein isolate/xanthan gum (WPI-X) gels prepared with 100 mM*NaCl*, and varying volume fraction filler (φ_f_). Glass microspheres of varying size were used as model fillers, which are denoted in the table.

φ_f_	4 μm	7–10 μm	45–90 μm	150–210 μm
0	139.68 ± 4.03	174.12 ± 9.49	179.44 ± 13.77	179.44 ± 13.77
0.025	134.34 ± 2.16	183.63 ± 5.42	198.60 ± 3.94	190.83 ± 21.24
0.05	110.77 ± 4.58	180.95 ± 5.90	211.58 ± 5.52	202.42 ± 19.88
0.10	85.17 ± 7.54	185.59 ± 10.67	281.77 ± 18.38	231.80 ± 18.57
0.15	64.57 ± 7.64	202.63 ± 20.09	380.05 ± 23.90	278.94 ± 19.34
0.20	67.12 ± 5.43	228.27 ± 11.92	549.74 ± 37.07	396.71 ± 18.60
0.33	144.38 ± 19.99	319.04 ± 47.68	1034.77 ± 27.23	702.25 ± 52.40
0.43	514.88 ± 64.65	498.87 ± 85.70	1279.09 ± 30.67	1036.31 ± 67.85
0.50	1489.09 ± 237.61	720.87 ± 29.60	1262.98 ± 13.84	1019.23 ± 49.14

**Table 3:** Elastic modulus of heat-set whey protein isolate/xanthan gum (WPI-X) gels prepared with 200 mM*NaCl*, and varying volume fraction filler (φ_f_). Glass microspheres of varying size were used as model fillers, which are denoted in the table.

φ_f_	4 μm	7–10 μm	45–90 μm	150–210 μm
0	204.31 ± 10.71	136.50 ± 6.30	11.35 ± 1.70	11.35 ± 1.70
0.025	158.63 ± 4.25	146.17 ± 1.40	13.51 ± 0.78	13.18 ± 2.60
0.05	154.68 ± 22.90	211.91 ± 24.04	22.43 ± 4.01	18.35 ± 4.34
0.10	154.95 ± 8.85	264.85 ± 1.56	41.44 ± 6.07	27.78 ± 4.56
0.15	62.67 ± 10.66	353.44 ± 13.02	82.19 ± 5.68	41.73 ± 9.44
0.20	98.37 ± 5.02	381.10 ± 13.53	193.17 ± 15.11	85.72 ± 7.60
0.33	210.93 ± 10.88	647.92 ± 28.70	735.08 ± 11.04	262.59 ± 17.62
0.43	552.67 ± 26.67	757.72 ± 156.04	1273.75 ± 54.58	682.67 ± 25.75
0.50	1485.09 ± 31.19	956.61 ± 79.98	1372.42 ± 101.41	1071.29 ± 71.47

Table 4 consists of an extended dataset highlighting the impact of increasing ionic strength on the composite gels filled with 4 μm glass beads. It is noted that in the absence of added salt (0 mM NaCl), the glass microspheres behaved as ideal bound fillers, in line with that expected by established particle reinforcement theories [3], [4]. The trends in E_c_ with increasing NaCl content indicate that the addition of salt progressively weakens the protein/filler interfacial interaction, causing a reduction in the interfacial adhesion. Although this point represents an interpretation of experimental data, it is highly relevant to the proposed application of theoretical models.

**Table 4:** Elastic modulus of heat-set whey protein isolate/xanthan gum (WPI-X) gels prepared with 4 μm*glass microspheres* as model fillers (denoted as volume fraction filler, φ_f_) and varying ionic strength. Ionic strength was varied via addition of NaCl, which is denoted in the table.

φ_f_	0 mM	50 mM	100 mM	200 mM
0	6.95 ± 0.46	78.01 ± 0.76	139.68 ± 4.03	204.31 ± 10.71
0.025	10.44 ± 0.43	61.15 ± 1.61	134.34 ± 2.16	158.63 ± 4.25
0.05	12.45 ± 1.11	41.63 ± 0.97	110.77 ± 4.58	154.68 ± 22.90
0.10	27.22 ± 1.33	41.42 ± 0.66	85.17 ± 7.54	154.95 ± 8.85
0.15	30.01 ± 0.23	44.43 ± 1.01	64.57 ± 7.64	62.67 ± 10.66
0.20	46.52 ± 4.67	50.43 ± 5.62	67.12 ± 5.43	98.37 ± 5.02
0.33	128.58 ± 13.24	131.06 ± 5.28	144.38 ± 19.99	210.93 ± 10.88
0.43	436.86 ± 2.21	432.96 ± 22.18	514.88 ± 64.65	552.67 ± 26.67
0.50	1611.18 ± 62.75	1654.65 ± 114.99	1489.09 ± 237.61	1485.09 ± 31.19

Note: All data tables are also provided in an Excel worksheet as supplementary data.

## 2 Experimental design, materials, and methods

### 2.1 WPI-X composite gel preparation

Stock biopolymer solutions were prepared in 200 ml batches by mixing 10 wt% whey protein isolate (BiPro™ USA, 91% protein) in deionized water for a minimum of 30 min using a magnetic stir bar. Xanthan gum (0.85 wt%; TIC Gums, Inc.) was subsequently added as a thickening agent to avoid sedimentation of the filler particles. The solutions were then mixed for a minimum of 2 hrs to allow full dissolution. The stock was then split into 100 ml portions, and NaCl was added to adjust the ionic strength (0 mM, 50 mM, 100 mM, or 200 mM), followed by mixing for several minutes. Individual samples were prepared in 30 g batches with varying filler content (φ_f_ = 0, 0.025, 0.05, 0.10, 0.15, 0.20, 0.33, 0.43, and 0.50), and glass beads were incorporated by hand mixing. Due to a small amount of alkaline residue present on the glass particles, the final pH of the particle-filled gels was ∼7.5. Control samples were therefore pH corrected to 7.5 using 0.5 N NaOH. For each sample, two 10 ml disposable polypropylene beakers (Fisher Scientific) were filled, covered with plastic food wrap to minimize evaporation, and placed in a metal test-tube rack. Samples were then heated in batches for 30 min in a water bath (Haake W-26, Haake Technik, GmbH) maintained at 80 °C. Upon removal from the water bath, samples were placed in a refrigerator a 4 °C for a minimum of 2 hrs prior to mechanical testing. All samples were prepared in triplicate.

### 2.2 Large deformation mechanical analysis

To evaluate the elastic modulus of the composite gels, uniaxial compression test were performed immediately after removal from the refrigerator. Prior to analysis, each sample was removed from the disposable beaker, and prepared by trimming the base flush and cutting to a height of 10 mm using a custom-made cutting block. Due to the slight conical shape of the beakers, the average diameter of the composite gels was 22 mm. Large deformation tests were performed using a uniaxial compression test [5], in which each sample was subjected to a compression strain of 60% at a rate of 0.83 mm/sec. Compression tests were performed using a texture analyzer (model TA.XT2, Stable Micro Systems, Ltd.) outfitted with a 100 mm stainless steel compression plate and a 30 kg load cell. A total of 6 measurements were performed for each formulation; 2 beakers per specimen, each prepared independently in triplicate. Data was collected and analyzed using the Exponent Lite Express and Exponent software, respectively (Stable Micro Systems).

The apparent elastic constant was determined by taking the slope of the linear region within the initial stage of compression from the true stress vs true strain curves, such that the strain was between 1 and 10% (i.e. a true strain of 0.01–0.1) [6]. The apparent elastic modulus was then determined based on the geometry of the sample, and is reported in units of kPa.
