# Supplementary material for: Dataset on the elastic modulus of heat-set whey protein isolate/xanthan gum mixed biopolymer hydrogels filled with glass microspheres: A model particle-filled composite food system
Source: Data Brief. 2019 May 27;25:104066. doi: 10.1016/j.dib.2019.104066 (PMC6580114; doi:10.1016/j.dib.2019.104066)
Supplement: Supplementary file 1 — Multimedia component 1 [file mmc1.pdf]

Conflict of Interest Statement and Authorship Conformation

I wish to confirm that I am one author signing on behalf of all co-authors of the manuscript. It is the understanding of all authors that in my role of Corresponding Author, I am the sole contact for the Editorial process (including Editorial Manager and direct communications with the office). I am responsible for communicating with the other authors about progress, submissions of revisions and final approval of proofs. All authors have provided a current, correct email address which is accessible through the Corresponding Author, and which has been configured in the email from the Data in Brief Journal.

I confirm that there are no known conflicts of interest associated with this publication and there has been no significant financial support for this work that could have influenced its outcome.

I confirm that the manuscript has been read and approved by all named authors and that there are no other persons who satisfied the criteria for authorship but are not listed. I further confirm that the order of authors listed in the manuscript has been approved by all authors. This manuscript has not been submitted to, nor is under review at, another journal or other publishing venue.

I confirm that we have given due consideration to the protection of intellectual property associated with this work and that there are no impediments to publication, including the timing of publication, with respect to intellectual property. In so doing, we have followed the regulations of our institutions concerning intellectual property.

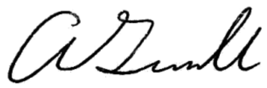

Andrew J. Gravelle
Corresponding Author
